# Targeting SOD1 induces synthetic lethal killing in *BLM- and CHEK2*-deficient colorectal cancer cells

**DOI:** 10.18632/oncotarget.4875

**Published:** 2015-07-31

**Authors:** Babu V. Sajesh, Kirk J. McManus

**Affiliations:** ^1^ Department of Biochemistry and Medical Genetics, University of Manitoba, Winnipeg, Manitoba, Canada; ^2^ Research Institute of Oncology and Hematology, Winnipeg, Manitoba, Canada

**Keywords:** BLM, CHEK2, SOD1, synthetic lethality, drug targeting

## Abstract

Cancer is a major cause of death throughout the world, and there is a large need for better and more personalized approaches to combat the disease. Over the past decade, synthetic lethal approaches have been developed that are designed to exploit the aberrant molecular origins (i.e. defective genes) that underlie tumorigenesis. *BLM* and *CHEK2* are two evolutionarily conserved genes that are somatically altered in a number of tumor types. Both proteins normally function in preserving genome stability through facilitating the accurate repair of DNA double strand breaks. Thus, uncovering synthetic lethal interactors of *BLM* and *CHEK2* will identify novel candidate drug targets and lead chemical compounds. Here we identify an evolutionarily conserved synthetic lethal interaction between *SOD1* and both *BLM* and *CHEK2* in two distinct cell models. Using quantitative imaging microscopy, real-time cellular analyses, colony formation and tumor spheroid models we show that SOD1 silencing and inhibition (ATTM and LCS-1 treatments), or the induction of reactive oxygen species (2ME2 treatment) induces selective killing within *BLM*- and *CHEK2*-deficient cells relative to controls. We further show that increases in reactive oxygen species follow SOD1 silencing and inhibition that are associated with the persistence of DNA double strand breaks, and increases in apoptosis. Collectively, these data identify SOD1 as a novel candidate drug target in *BLM* and *CHEK2* cancer contexts, and further suggest that 2ME2, ATTM and LCS-1 are lead therapeutic compounds warranting further pre-clinical study.

## INTRODUCTION

Colorectal cancer (CRC) is a major cause of cancer-related deaths worldwide. In 2015, the American Cancer Society estimates that ~132,700 Americans will be newly diagnosed and ~50,000 additional individuals will succumb to the disease in 2015 [1]. These statistics highlight the need for novel personalized therapeutic strategies designed to better combat the disease. Synthetic lethality is one such strategy, and is defined as the lethal combination of two independently viable mutations. In a cancer context, a cancer-driving mutation is leveraged to lethality through the down-regulation (i.e. silencing or inhibition) of a synthetic lethal (SL) interactor (i.e. drug target) [2]. Thus, synthetic lethality exploits the causative genetic aberrations synonymous with tumor development and progression. Accordingly, identifying SL interactors of genes somatically altered in cancer will uncover novel candidate drug targets whose inhibitors represent lead therapeutic agents.

Recent gene re-sequencing efforts have uncovered a myriad of somatic mutations and deletions in genes associated with DNA double strand break (DSB) repair pathways [3, 4]. Somatic alterations of these cancer genes are correlated with genome instability (i.e. DNA damage and chromosome instability) [5], which contributes to the acquisition of subsequent mutations, some of which confer growth advantages that can enhance tumor growth and metastasis. For example, *BLM* and *CHEK2* are somatically altered in a number of tumor types including CRC [3, 4, 6], and normally function within the homology directed repair (HDR) pathway (“error-proof” DSB repair pathway). More specifically, BLM is a member of the RECQ helicase family, and harbors ATP-dependent 3′-5′ DNA helicase activity (reviewed in [7]), which is required for HDR [7–12]. In addition, germline mutations in *BLM* are pathogenic for Bloom syndrome, an inherited disorder associated with an increased predisposition to develop many tumor types including CRC [13]. CHEK2 is a tumor suppressor that regulates genome stability [14]. It normally functions in HDR by inducing cell cycle checkpoints so that DSBs can be accurately repaired [15–18]. Thus, aberrant CHEK2 activity is associated with checkpoint defects, inadequate DNA repair, and cancer development. Accordingly, identifying novel strategies and candidate drug targets capable of exploiting genetic defects in *BLM* and *CHEK2* are highly warranted.

In this study, we couple siRNA-based silencing and chemical compounds with semi-quantitative imaging microscopy, real time cellular analyses (RTCA), and biochemical assays to show that *BLM* and *CHEK2* are SL with *SOD1*. We demonstrate that *BLM*- and *CHEK2*-deficient CRC cells are selectively killed following *SOD1* silencing and recapitulate these findings within an additional and unrelated cellular content. We further show that two SOD1 inhibitors (ammonium tetrathiomolybdate [ATTM] and Lung Cancer Screen-1 [LCS-1]) and one chemical mimetic (2-methoxyestradiol [2ME2]) phenocopy the SOD1 silencing results by inducing preferential killing within *BLM-* and *CHEK2-*deficient cells. Using semi-quantitative microscopy and RTCA, we show that all three chemicals induce reactive oxygen species (ROS), promote persistent DNA DSBs and potentiate cellular cytotoxicity through an apoptotic mechanism. Finally, we show that drug treatments significantly decrease the number and size of *BLM*- and *CHEK2*-deficient cells in 2D colony and 3D tumor spheroid formation assays, respectively. Collectively, our data shows that BLM and CHEK2 are SL with SOD1, and further identify 2ME2, ATTM and LCS-1 as lead candidate compounds warranting further pre-clinical study.

## MATERIALS AND METHODS

### Cell culture

HCT116 (*BLM*-proficient and *CHEK2*-proficient) cells were purchased from American Type Culture Collection. *BLM*-deficient and *CHEK2*-deficient, HCT116 cells were generously provided by Dr. Bert Vogelstein (Johns Hopkins University, Baltimore). All HCT116 cells were grown in McCoy's 5A medium (HyClone) containing 10% fetal bovine serum. Immortalized (telomerase) BJ normal human skin fibroblasts, hTERT, were generously provided by C.P. Case [19] (University of Bristol, Bristol, UK) and were grown in DMEM supplemented with 10% fetal bovine serum. Cells were authenticated based on the recovery, viability, growth and morphology, while parental HCT116 and hTERT cells were also authenticated by spectral karyotyping as detailed elsewhere [20]. BLM and CHEK2 expression was confirmed in all cell lines by Western blots. All cells were grown in a 37°C humidified incubator with 5% CO_2_.

### Gene silencing

Cells were transiently transfected with siRNA duplexes using RNAiMax (Invitrogen) as described [20]. ON-TARGETplus (Dharmacon) siRNA duplexes targeting SOD1, BLM, CHEK2, GAPDH and PLK1 were employed as either individual duplexes or pools (four distinct duplexes targeting the gene of interest), as detailed previously [20]. Gene silencing was confirmed by Western blots using the antibodies and dilutions indicated in Supplementary Table 1.

### Direct SL tests

High-content microscopy was used to evaluate the SL interactions as detailed elsewhere [20]. Briefly, 8,000 *BLM-*deficient, *CHEK2-*deficient and control (HCT116) cells, and 4,000 hTERT cells were automatically dispensed into each well of a 96-well plate (BioTek; EL406). Cells were transfected in sextuplet (i.e. 6-wells) with either individual or pooled siRNAs targeting BLM, CHEK2, SOD1, and controls (GAPDH and PLK1) as described [20]. Cells were permitted to grow for 4- (HCT116) or 5-days (hTERT) following which cells were fixed (4% paraformaldehyde), and counterstained with Hoechst 33342 (Sigma; 0.2 μM). Images were acquired using a Cytation 3 (BioTek) equipped with a 10× objective (0.3 numerical aperture), a 16-bit gray scale charged couple device camera, and GEN5 software. Twelve central and non-overlapping images were acquired per well (condition), and the total number of cells in each well and condition were determined. All data were imported into Prism v6.0 (GraphPad), normalized to GAPDH silenced controls, and basic statistical analyses (e.g. mean, standard deviation, Student's *t*-tests, etc.) were performed. To address reproducibility all experiments were conducted a minimum of 3-times.

### Dose response curves

Standard dose response curves were generated as detailed previously [20] using a 10-fold (100pM to 1M) serial dilution for 2ME2, ATTM, or LCS-1 [21]. Briefly, ~8,000 cells were seeded into each well of a 96-well plate, permitted to attach, and growth medium supplemented with appropriate concentrations of 2ME2, ATTM, LCS-1 or vehicle control (DMSO) were added to wells in sextuplet. Following 3-days of growth, cells were fixed, counterstained, imaged and analyzed as above. All data were normalized to DMSO-treated controls and half maximal effective concentrations (EC_50_) values were determined. The EC_50_ values calculated from the *BLM*- and *CHEK2*-deficient cells were employed in all subsequent experiments.

### ROS detection

ROS were detected using the Image-IT LIVE Green ROS detection kit (Molecular Probes) as detailed elsewhere [20] with minor modifications. Briefly, 8,000 cells were seeded into each well of a 96-well plate, and permitted to attach. The following day, SOD1 or GAPDH were silenced in control, *BLM*- and *CHEK2*-deficient cells for 48 h or cells were treated with compounds (2ME2, ATTM, LCS-1 and DMSO) for 6 h. Each condition was performed in sextuplet and repeated two additional times. Images were acquired (Cytation 3), signal intensities were determined from raw, unprocessed images (GEN5), and semi-quantitative analyses were performed as described [20, 22]. All data were imported into Prism, normalized to untreated controls, and basic statistical analyses (e.g. mean, standard deviation, Student's *t*-tests, etc.) were performed.

### Quantitative imaging microscopy

Semi-quantitative imaging microscopy was employed to evaluate the presence of DNA DSBs as represented by the total signal intensities of two surrogate markers, namely γ-H2AX and 53BP1, as described previously [20, 22]. Briefly, 8, 000 cells were seeded into each well of a 96-well plate, permitted to attach, and grow for 48 h. Next, cells were treated with Bleomycin (0.1 μg/mL) for 2 h, DMSO for 42 h, or 2ME2, ATTM and LCS-1 for 6 h. The media from a subset of wells containing 2ME2, ATTM, and LCS-1 was aspirated, washed once with pre-warmed phosphate buffered saline (pH 7.4), replaced with fresh growth media lacking 2ME2, ATTM and LCS-1, and cells were permitted to grow for an additional 36 h. Following washout and recovery, cells were processed for semi-quantitative microscopy as detailed [20], and images were acquired as described above (*Direct SL Tests*). Each condition was performed in sextuplet and repeated at least two additional times with γ-H2AX and 53BP1 signal intensities determined from raw, unprocessed images. Apoptosis was evaluated by quantifying the total signal intensity of cleaved Caspase 3 as described previously [20]. All data were imported into Prism and were normalized to the appropriate negative or vehicle control. Antibodies and working dilutions are presented in Supplementary Table 1.

### Real time cellular analyses

RTCA were performed in quadruplicate and repeated twice using an xCELLigence RTCA-dual plate (DP) system (Acea Biosciences) as detailed [20]. Approximately 8,000 cells were seeded into each well of an E-Plate (Acea Biosciences) and growth (electrical impedance) was measured every 10 minutes. DMSO, 2ME2, ATTM, or LCS-1 were supplemented into growth medium when cells attained ~25% of their untreated maximal values (~48 h post seeding) and growth was monitored for up to 7-days. All data were imported into Prism and growth curves were plotted for each condition of treatment.

### ROS scavenging/chemical rescue

N-acetyl-L-cysteine (NAC) was used to scavenge excessive ROS. The maximum tolerated dose of NAC was determined for *BLM-*deficient (2 μM) and *CHEK2-*deficient (5 μM) cells by RTCA. RTCA was performed as above, but with media supplemented with DMSO, 2ME2, ATTM or LCS-1 with or without NAC. All data were imported into Prism where growth curves were plotted.

### Colony formation and 3D tumor spheroid assays

The effect of prolonged DMSO, 2ME2, ATTM and LCS-1 treatments was determined using a standard 28-day colony forming assay as described [20], and tumor spheroid assays as directed by the manufacturer (InSphero). In brief, 1,000 *BLM*- or *CHEK2*-deficient, or control cells were dispensed into GravityPLUS plates, and tumors were permitted to form for 2-days, whereupon they were released into GravityTRAP plates and cultured in growth medium containing DMSO, 2ME2, ATTM, or LCS-1. Media containing DMSO, 2ME2, ATTM, or LCS-1 were replaced every 2- to 3-days. Cells were incubated for 14-days, at which point nuclei were counterstained with Hoechst 33342, and the medial plane of each tumor spheroid was imaged using a Cytation 3 (10× objective; NA = 0.13). ImageJ was employed to determine the maximal diameter of the spheroid [23]. All data were imported into Prism, normalized to the appropriate DMSO-treated control, and Student's *t*-tests were performed. Finally, all experiments were performed in sextuplet and repeated at least two additional times.

## RESULTS

### BLM and CHEK2 are synthetic lethal with SOD1

To identify novel and lead candidate drug targets to evaluate in a CRC context, we recently employed a cross-species candidate gene approach and identified the SL interactors of yeast genes whose human orthologs are somatically altered in CRC. We queried BioGRID [24] and identified all SL interactions for the 692 yeast chromosome instability genes [20]. As an entry point, two-dimensional hierarchical clustering was performed [20], and a collection of 30 genes each harboring > 22 SL interactors were identified. This collection was highly enriched for genes encoding functions within DSB repair, particularly HDR including *sgs1* and *dun1*, which are the yeast orthologs of human *BLM* and *CHEK2*, respectively. Interestingly, *sgs1* and *dun1* are SL with several members of the evolutionarily conserved superoxide dismutase pathway, including superoxide dismutase-1 (yeast *sod1*/human *SOD1*). This pathway normally functions to remove excess ROS through a two-step process initially regulated by SOD1, which requires Cu^2+^ as an essential co-factor [25, 26]. Excessive ROS induce a variety of cellular damage including DSBs, which we presumed would not be accurately repaired in HDR-defective cells (e.g. *BLM* and *CHEK2*-deficient), and would underlie cell cytotoxicity. Accordingly, SOD1 represents a strong candidate to pursue in a cross-species approach designed to identify novel drug targets (i.e. SL interactors) for *BLM* and *CHEK2*.

**Figure 1:**
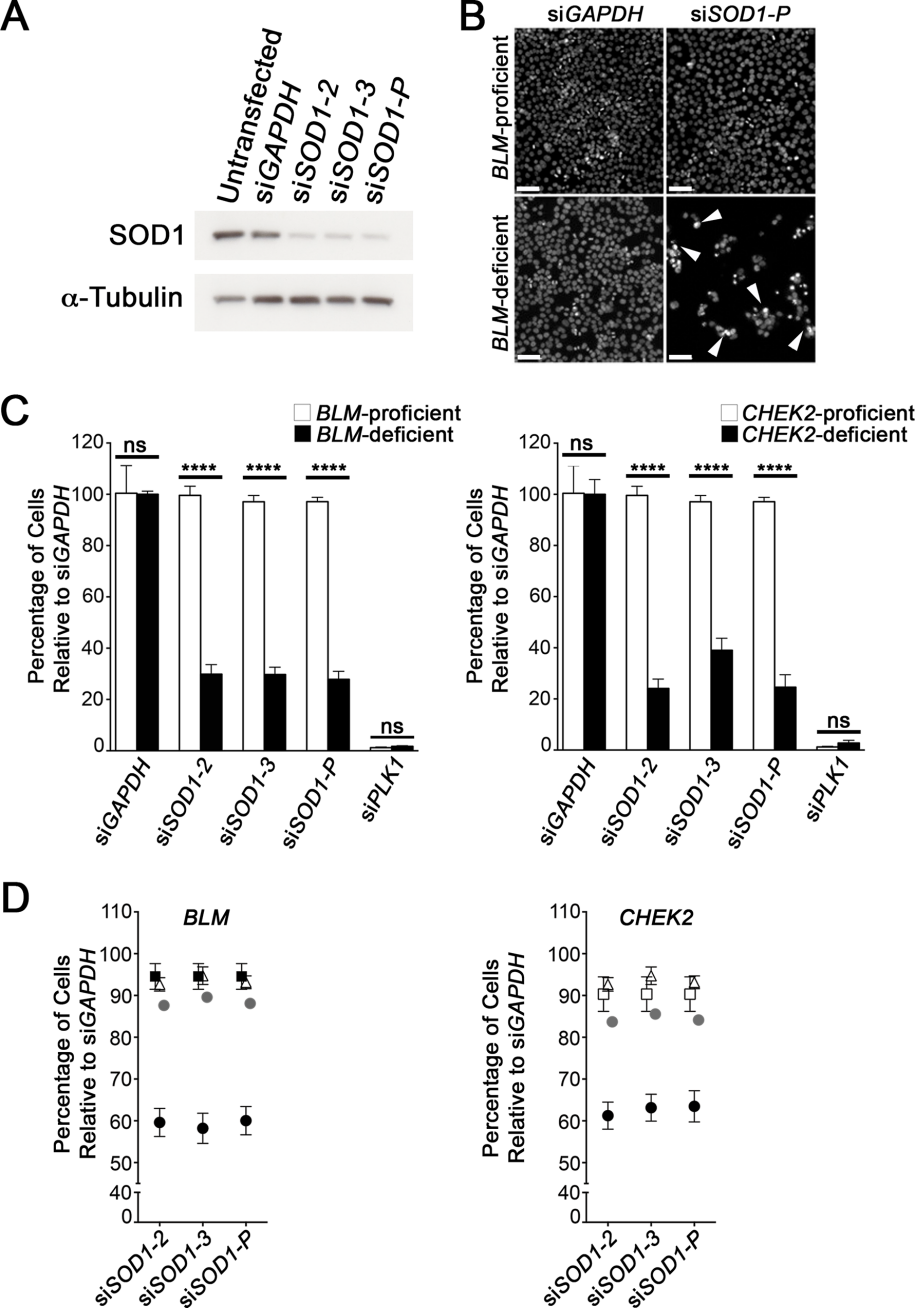
*BLM* and *CHEK2* are synthetic lethal with *SOD1* **A.** Western blot depicting SOD1 silencing in HCT116 cells with either individual (siSOD1–2 and siSOD1–3) or pooled (siSOD1-P) siRNA duplexes relative to controls (untransfected and siGAPDH); α-Tubulin serves as a loading control. **B.** Representative low-resolution images depicting the decrease in Hoechst stained nuclei (bottom right quadrant) following SOD1 silencing in *BLM*-deficient cells. Arrowheads identify nuclei exhibiting apoptotic hallmarks. Scale bars represent 100 μm. **C.** Graph depicting the statistically significant decrease in *BLM*- (left) or *CHEK2*-deficient cells (right) following SOD1 silencing relative to controls. The statistical significance is indicated (*ns*, not significant; ****, *p*-value < 0.0001). *GAPDH* serves as the negative control, while *PLK1* is an essential gene used as a positive control for death and a transfection indicator. **D.** Graphs depicting the SL interaction observed following simultaneous silencing of BLM (left) or CHEK2 (right) with SOD1 in HCT116 cells. Presented are the mean normalized percentages (± SD) for the individual silencing of either BLM (solid squares) or CHEK2 (open squares) and SOD1 (open triangles), and the expected value (grey circles) determined for the dual combined siRNAs as calculated using a multiplicative model. Solid circles identify the actual observed values for the simultaneous dual silencing (i.e. BLM and SOD1, or CHEK2 and SOD1) and are lower than the expected values indicating a SL phenotype.

In budding yeast, *sgs1* and *dun1* are SL with *sod1* [27]. To determine whether *SOD1* is SL with *BLM* and *CHEK2* in humans, we employed an established siRNA-based approach [20, 28]. Briefly, *BLM*-deficient (or *CHEK2*-deficient) and control HCT116 (*BLM*- and *CHEK2*-proficient) cells are silenced with either individual or pooled siRNAs targeting SOD1 and controls (GAPDH), and the total number of cells remaining is statistically compared. A SL interaction is expected to result in fewer cells within the deficient cell lines. Before performing the SL tests, we first confirmed BLM and CHEK2 expression levels within all cell lines by Western blots (Supplementary Figure 1), and evaluated the silencing efficiency of two individual (siSOD1–2 and siSOD1–3) and pooled (siSOD1-P) siRNA duplexes (Figure 1A). Next, we performed direct SL tests and as predicted there were visually striking decreases in the number of *BLM-* and *CHEK2*-deficient cells relative to controls (Figure 1B) that quantitative imaging microscopy determined to be statistically significant (Figure 1C and Supplementary Table 2). Further scrutiny of the images revealed a population of *BLM*- and *CHEK2*-deficient cells exhibiting hallmarks of apoptosis including nuclear blebbing and increased chromatin condensation that were not readily apparent within the controls (Figure 1B). Collectively, these data suggest that SOD1 silencing selectively induces cytotoxicity within *BLM*- and *CHEK2*-deficient cells, and further suggest that these genetic interactions are evolutionarily conserved.

Although the above observations suggest *BLM* and *CHEK2* are SL with *SOD1*, it remains possible that the putative interactions are due to background mutations that accrued during the generation of the *BLM-* and *CHEK2*-deficient cells. To assuage this possibility, dual siRNA experiments (e.g. BLM plus SOD1 silencing) were performed within the parental, HCT116 cells. In agreement with the above data, Figure 1D shows that the simultaneous silencing of either *BLM* or *CHEK2* with *SOD1* resulted in fewer cells than each condition alone, or the expected number as calculated by a multiplicative model (Supplementary Table 3). The percentage of cells remaining was similar and ~60% with either the individual or pooled approaches for both BLM and CHEK2. Although the total decrease in cell numbers was not as large as with the *BLM*- and *CHEK2*-deficient cells employed above, we attribute this to the residual proteins remaining following siRNA-based silencing compared with the their complete absence within the *BLM*- and *CHEK2*-deficient cells (Supplementary Figure 2).

To extend these findings beyond the HCT116 context employed above, analogous dual siRNA-based experiments were performed in hTERT, a karyotypically stable and immortalized fibroblast cell line. Western blots were first performed to confirm silencing efficiencies (Supplementary Figure 3), and in agreement with the HCT116 findings, dual siRNA-based silencing (siBLM/siCHEK2 and siSOD1) resulted in fewer hTERT cells than expected using a multiplicative model (Supplementary Figure 4 and Supplementary Table 4). Collectively, these data support the evolutionarily conserved SL interaction between *BLM* or *CHEK2* and *SOD1*, and further demonstrate that these interactions are independent of cell type.

### 2ME2, ATTM and LCS-1 induce selective Killing in BLM- and CHEK2-deficient cells in a ROS-dependent manner

**Figure 2:**
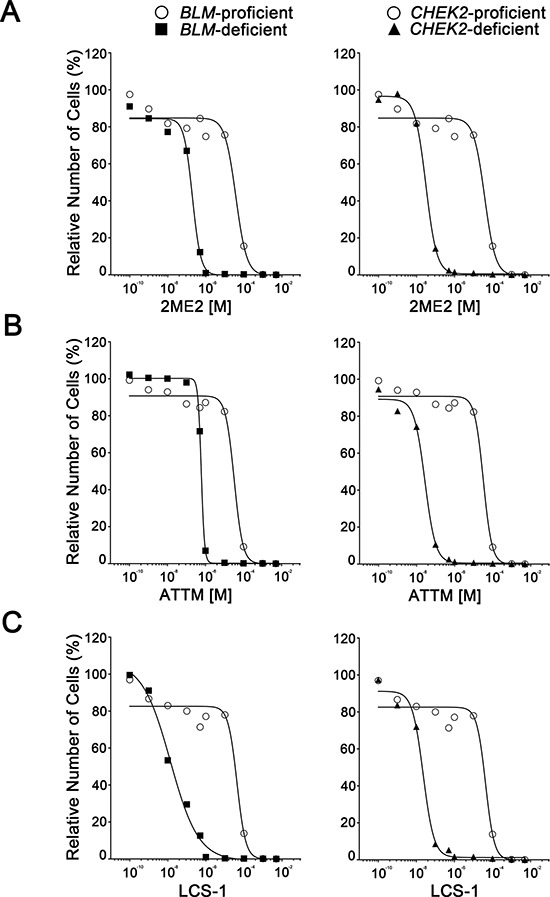
*BLM*- and *CHEK2*-deficient cells are hypersensitive to 2ME2, ATTM and LCS-1 Standard dose response curves for cells (indicated at top) treated with varying concentrations of 2ME2 **A.** ATTM **B.** and LCS-1 **C.** Data are normalized to the respective DMSO-treated controls.

Having established that *BLM* and *CHEK2* are SL with *SOD1*, we now wished to determine if three chemical compounds, 2ME2, ATTM and LCS-1 predicted to phenocopy SOD1 silencing could substitute for the siRNAs and induce death specifically within the *BLM*- and *CHEK2*-deficient cells. While 2ME2 induces ROS including superoxide anions [29], ATTM and LCS-1 are a Cu^2+^ chelator [26] (required for SOD1 activity) and a SOD1 inhibitor [21] respectively. Prior to evaluating the SL interactions, standard dose response curves were generated for each compound within the *BLM*- and *CHEK2*-deficient, and control cells. As predicted, the *BLM*- and *CHEK2*-deficient cell lines are hypersensitive to each compound relative to controls (Figure 2). In addition, increases in cells exhibiting apoptotic hallmarks were also observed within the *BLM*- and *CHEK2*-deficient cells following treatments. Table 1 presents the EC_50_ (effective concentration at which 50% of the cells remain relative to DMSO-treated controls) values calculated for the *BLM*- and *CHEK2*-deficient cells relative to controls. In support of chemogenetic SL interactions, the EC_50_ values for 2ME2, ATTM and LCS-1 are approximately 193-, 54- and 3, 150-fold lower, respectively, in *BLM*-deficient cells, while they are approximately 1,230-, 1,175- and 1,892-fold lower in *CHEK2*-deficient cells, respectively. Finally, a fluorogenic ROS indicator coupled with semi-quantitative imaging microscopy and was employed to confirm that 2ME2, ATTM and LCS-1 treatments each induce ROS formation (Supplementary Figure 5), which also occurs following SOD1 silencing (Supplementary Figure 6).

**Table 1:** EC_50_ values calculated from dose response curves

Compound	HCT116 (control) EC_50_ (nM)	*BLM*-deficient EC_50_ (nM)/FI^A^	*CHEK2*-deficient EC_50_ (nM)/FI^A^
2ME2	3.82 × 10^4^	1.98 × 10^2^/193	3.10 × 10^1^/1232
ATTM	3.23 × 10^4^	5.98 × 10^2^/54	2.75 × 10^1^/1175
LCS-1	4.31 × 10^4^	1.37 × 10^1^/3145	2.28 ×10^1^/1889

A FI; Fold increase in sensitivity relative to the corresponding HCT116 control.

**Figure 3:**
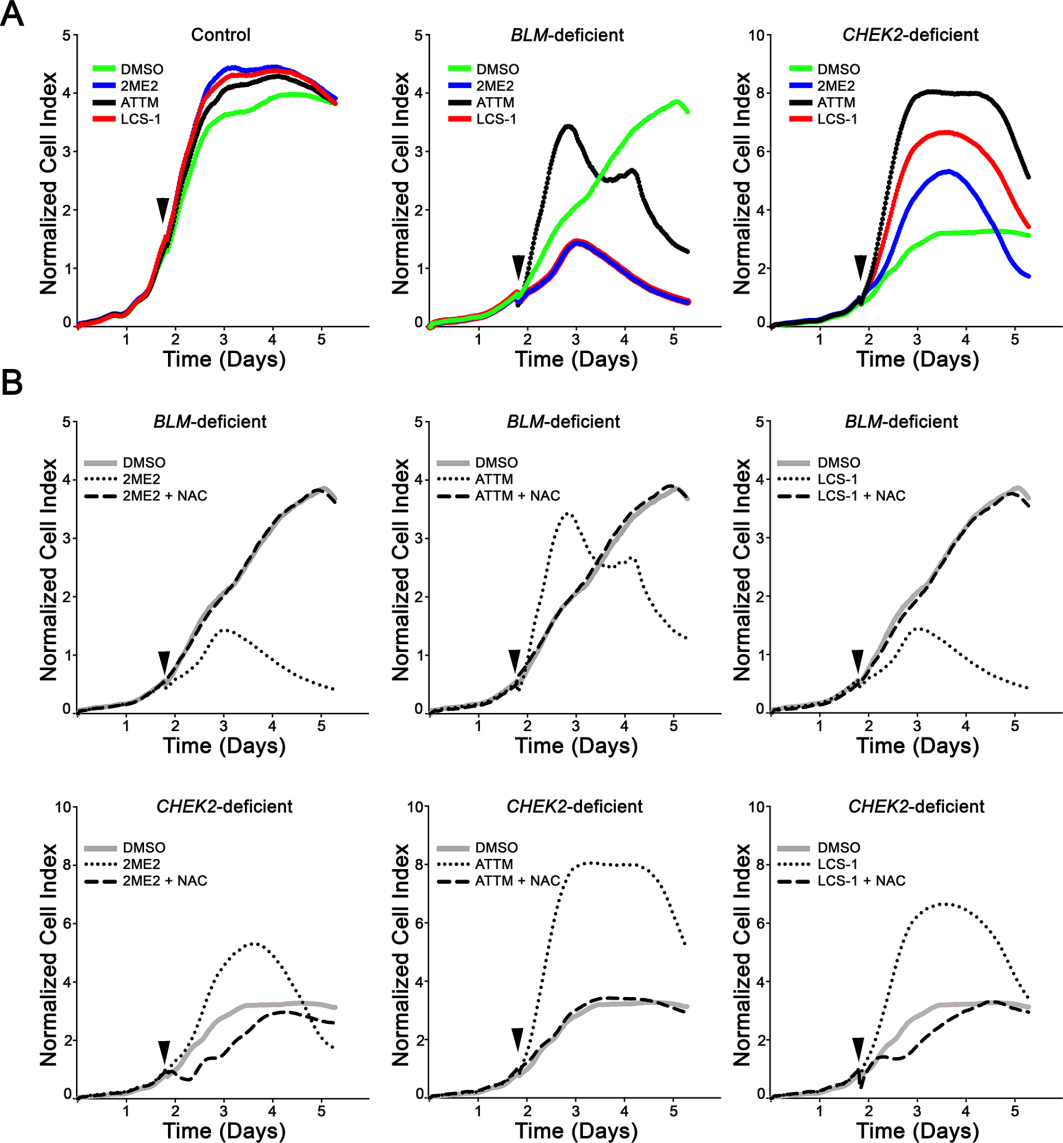
NAC administration rescues the hypersensitivity of *BLM*-deficient and *CHEK2*-deficient cells to 2ME2, ATTM and LCS-1 **A.** RTCA growth curves for control (left), *BLM*- (middle) and *CHEK2*-deficient (right) cells treated with DMSO, 2ME2, ATTM or LCS-1. Arrowheads identify the timepoints at which the chemicals were administered. **B.** RTCA depicting NAC rescue of *BLM*- (top panels) or *CHEK2*-deficient cells (bottom panels) treated with 2ME2 (left), ATTM (middle) or LCS-1 (right). Arrowheads identify the timepoints at which the chemicals were administered. Note that NAC addition restores growth back to approximately that of the corresponding DMSO-treated controls for all three compounds and in both cell lines.

To determine if the diminished cell numbers were due to cell cycle arrest or cellular cytotoxicity RTCA (i.e. growth curves) were performed. RTCA employs electrical impedance as a measure of cellular proliferation and can easily discern altered growth rates from cell cycle arrests and cellular cytotoxicity. To restrict the therapeutic effect to *BLM*- and *CHEK2*-deficient cells, we specifically employed the EC_50_ values determined for those cells. As predicted, the growth curves generated for the control cells (Figure 3A) were virtually indistinguishable irrespective of treatment (e.g. 2ME2, ATTM, LCS-1 and DMSO). However, there was considerable variation in the growth curves generated within the *BLM*- and *CHEK2*-deficient cells (Figure 3A), with each showing a rapid decline in cell index that is indicative of cell cytotoxicity. More specifically, within the *BLM*-deficient cells, 2ME2 and LCS-1 gave superimposable growth curves with a cell index marginally lower (0.5 a.u.) than that of DMSO treated controls, whereas ATTM treated cells had an increase in cell index (1.4 a.u.) approximately 1-day post-treatment. Approximately 2-days post-treatments, there was a rapid decline in the cell index in each of the condition of treatment that was not readily apparent in the DMSO treated controls. On the other hand, within the *CHEK2*-deficient cells, 2ME2, ATTM and LCS-1 treated cells showed a markedly high cell index (1.2, 5 and 3 a.u., respectively) when compared to DMSO treated controls in 24 hours of treatment with the chemical compounds, which is likely due to a temporary cell cycle arrest and flattening of the cell body (unpublished observation) causing an increase in cellular contacts and thus electrical impedance. Following a temporary arrest as evidenced by a plateau in cell index, there was a rapid and sharp decline in cell index that occurred approximately 2-days (2ME2), 3-days (LCS-1), or 4-days (ATTM) post-treatment. The rapid declines in cell indices for *BLM*- and *CHEK2*-deficient cells treated with 2ME2, ATTM and LCS-1 are indicative of strong cytotoxic effects, and are in agreement with the images exhibiting apoptotic hallmarks (see Figure 1B). These data further show that 2ME2, ATTM and LCS-1 have minimal impact on the growth of control cells indicating that the compounds and concentrations employed are selective to the *BLM*- and *CHEK2*-deficient cells.

To determine if oxidative stress (i.e. ROS generation) was required to induce the cytotoxicity observed above, NAC, an established ROS scavenger was employed in phenotypic rescue experiments. In all instances, NAC addition was sufficient to restore the growth of the *BLM*- and *CHEK2*-deficient cells treated with 2ME2, ATTM and LCS-1 to those of the vehicle control (Figure 3B). Collectively, these data indicate that 2ME2, ATTM and LCS-1 induce preferential cytotoxicity in the *BLM*- and *CHEK2*-deficient cells, and further show that ROS contributes to the cytotoxic effect.

### 2ME2, ATTM and LCS-1 induce persistent DNA DSBs in BLM- and CHEK2-deficient cells

**Figure 4:**
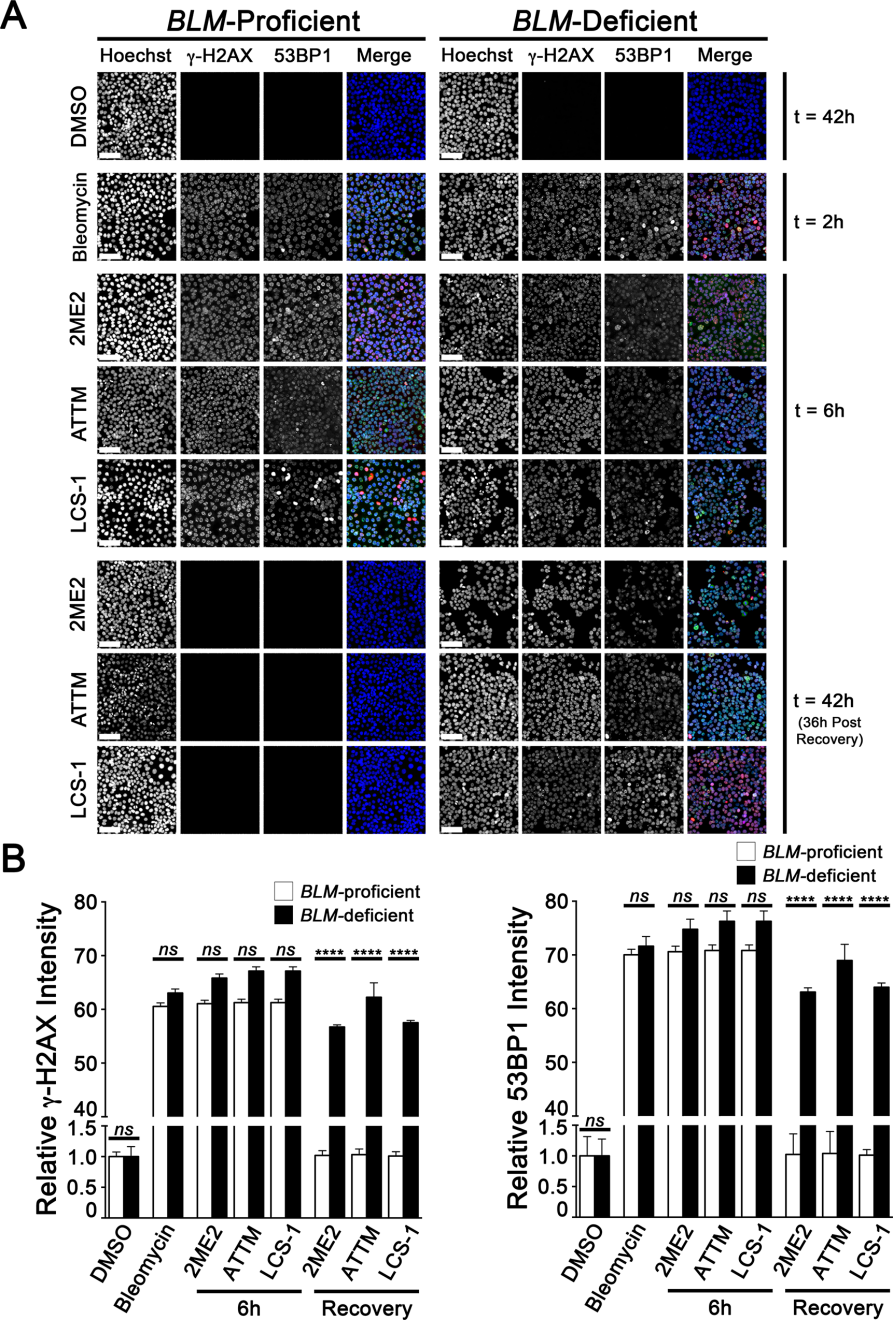
DNA DSBs persist in *BLM*-deficient cells treated with 2ME2, ATTM and LCS-1 **A.** Representative low-resolution (10×) images presenting the qualitative changes in γ-H2AX and 53BP1 signal intensities within control (left) and *BLM*-deficient cells (right) treated with DMSO, bleomycin (positive control), 2ME2, ATTM, or LCS-1. Cells were imaged after 2 h (t = 2 h; bleomycin) or 6 h (t = 6 h; DMSO, 2ME2, ATTM and LCS-1) treatments, or following treatment, washout and a 36 h recovery phase (t = 42 h). Nuclei were counterstained with Hoechst, and images were acquired using identical exposure times at each wavelength so that qualitative and quantitative analyses could be performed. Hoechst, γ-H2AX and 53BP1 are pseudo-colored blue, green, and red, respectively, within the merged images. Scale bars represent 100 μm. Note the persistence of γ-H2AX and 53BP1 signal intensities within the *BLM*-deficient cells following washout and recovery relative to controls. **B.** Graphs presenting the mean normalized γ-H2AX (left) and 53BP1 (right) signal intensities (± SD) within control and *BLM*-deficient cells treated with DMSO, bleomycin, 2ME2, ATTM, or LCS-1 or following washout and a 36 h recovery phase (t = 42 h). All data are presented relative to the DMSO-treated controls. Raw, unprocessed images were used to determine γ-H2AX and 53BP1 signal intensities. Note the persistence and statistically significant differences observed for γ-H2AX and 53BP1 following washout and recovery within the *BLM*-deficient cells relative to controls (ns, not significant; ****, *p*-value < 0.0001).

We now sought to determine the underlying mechanism contributing to the increases in cytotoxicity observed within the *BLM*- and *CHEK2*-deficient cells treated with 2ME2, ATTM and LCS-1. Since oxidative stress induces various types of cellular damage including DSBs [30–32], we reasoned that excessive DSBs caused by treatments would not be adequately repaired in HDR-defective cells, and would ultimately lead to cell cytotoxicity. Accordingly, the prevalence and persistence of DSBs was evaluated by semi-quantitative imaging microscopy at various time points using two well-established surrogate markers for DSBs, namely γ-H2AX and 53BP1. As expected, visually apparent and statistically significant increases in γ-H2AX and 53BP1 signal intensities within *BLM*- (Figure 4), *CHEK2*-deficient (Supplementary Figure 7) and control cells treated with 2ME2, ATTM and LCS-1 for 6 h relative to DMSO treated controls. However, following compound washout and a 36 h recovery phase, γ-H2AX and 53BP1 intensities remained elevated within the *BLM*- and *CHEK2*-deficient cells, but returned to basal levels within the controls. Thus, these data support the premise that 2ME2, ATTM and LCS-1 treatments induce ROS formation that promotes persistent DNA DSBs, which cannot be adequately repaired within the *BLM*- and *CHEK2*-deficient cells, and underlies the observed increases in cell cytotoxicity.

### 2ME2, ATTM and LCS-1 treatments induce apoptosis in BLM- and CHEK2-deficient cells

**Figure 5:**
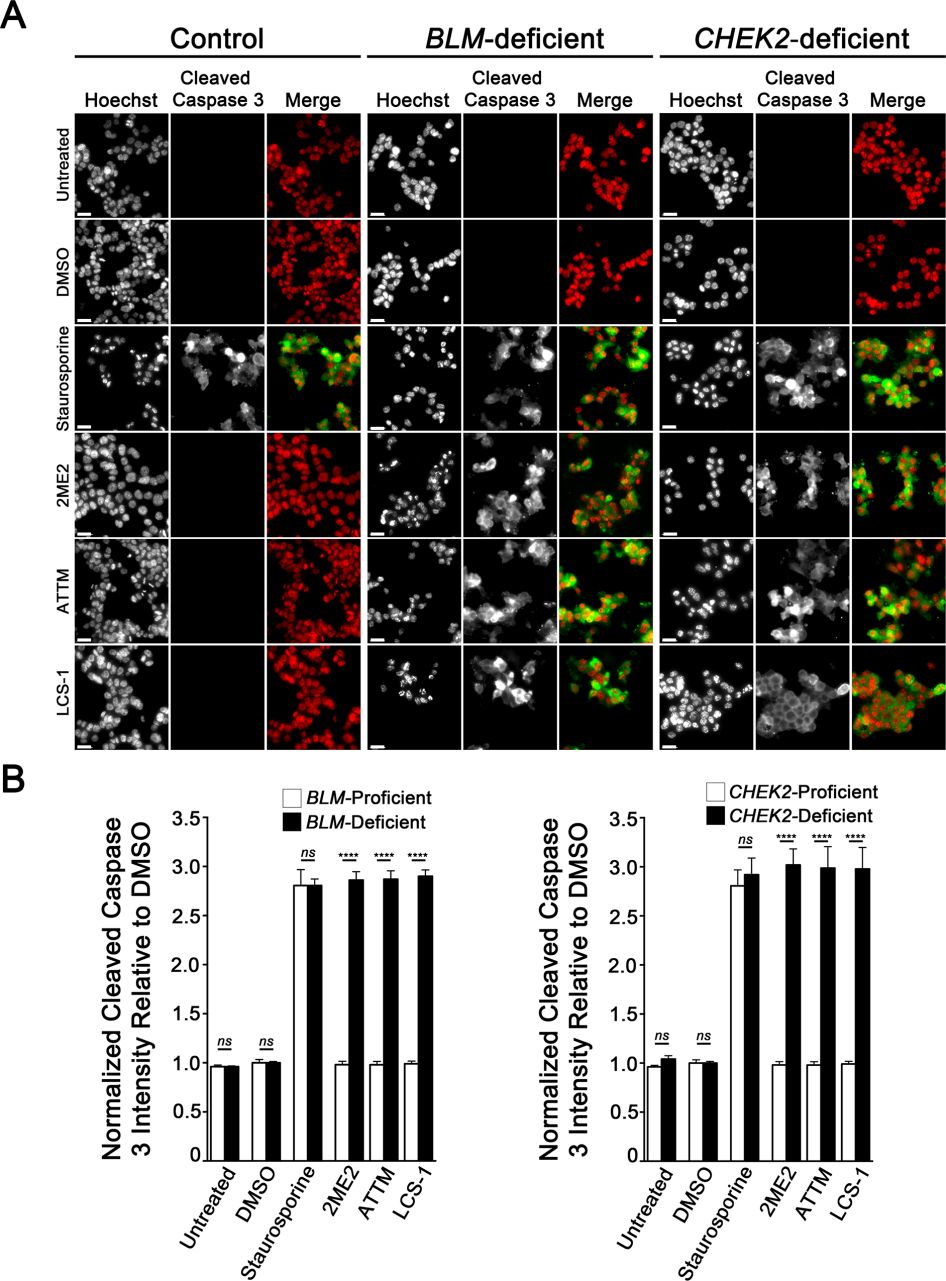
2ME2, ATTM and LCS-1 induce apoptosis in *BLM*- and *CHEK2*-deficient cells **A.** Representative low-resolution images (10×) presenting the qualitative differences in cleaved Caspase 3 signal intensities within control, *BLM*- and *CHEK*2-deficient cells treated with DMSO, staurosporine (positive control), 2ME2, ATTM and LCS-1. Cells were labeled for cleaved Caspase 3, while nuclei were counterstained with Hoechst. All images were collected using identical exposure times at each wavelength so that qualitative and quantitative analyses could be performed. Hoechst and cleaved Caspase 3 are pseudo-colored red and green, respectively within the merged images. Scale bars represent 30 μm. Note the visually striking increases in cleaved Caspase 3 signal intensities within the *BLM*- and *CHEK2*-deficient cells treated with 2ME2, ATTM and LCS-1 relative to controls. **B.** Bar graphs depicting the mean normalized cleaved Caspase 3 signal intensities (± SD) within control, *BLM*- (left) and *CHEK2*-deficient (right) cells treated with DMSO, staurosporine, 2ME2, ATTM and LCS-1. All data are presented relative to DMSO treated controls. Cleaved Caspase 3 signal intensities were determined from raw, unprocessed images. Note the statistically significant increase in cleaved Caspase 3 signal intensities within the *BLM*- and *CHEK2*-deficient cells relative to controls (ns, not significant; ****, *p*-value < 0.0001).

Although the above data show that *BLM*- and *CHEK2*-deficient cells are hypersensitive to 2ME2, ATTM and LCS-1 treatments, they do not address the underlying mechanism of death. However, recall that further scrutiny of images acquired following SOD1 silencing or compound treatments suggest apoptosis may be a contributing factor. Accordingly, we now wished to formally examine whether apoptosis contributes to the cytotoxicity observed within the *BLM* and *CHEK2* chemogenetic interactions identified above. Using semi-quantitative imaging microscopy and an antibody against cleaved Caspase 3, a key downstream apoptotic regulator, we evaluated apoptosis in cells treated with 2ME2, ATTM and LCS-1. As anticipated, *BLM*- and *CHEK2*-deficient cells treated with compounds exhibited statistically significant increases in the abundance of cleaved Caspase 3 relative to controls (Figure 5). More specifically, a 2.7- to 3.0-fold increase in DMSO-normalized cleaved Caspase 3 signal intensities occurred within the *BLM*- and *CHEK2*-deficient cells relative to controls. These data show that apoptosis contributes to the cytotoxicity observed following 2ME2, ATTM and LCS-1 treatments within the *BLM*- and *CHEK2*-deficient cells.

### 2ME2, ATTM and LCS-1 impair growth of BLM- and CHEK2-deficient cells in 3D cultures

**Figure 6:**
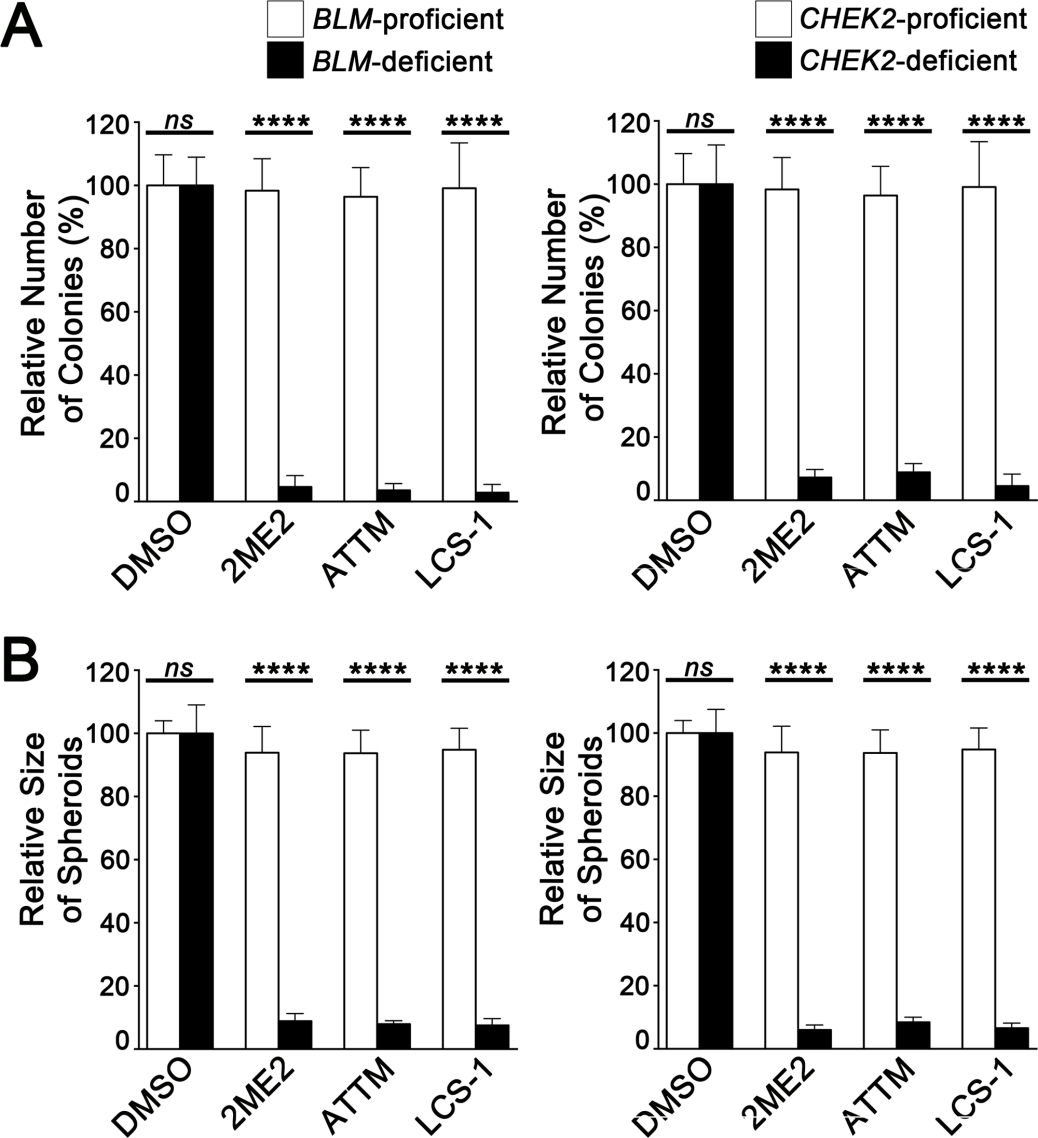
2ME2, ATTM and LCS-1 inhibit growth of 2D and 3D cultures of BLM- and CHEK2-deficient cells **A.** Bar graphs depicting statistically significant decreases in the mean number of *BLM*- and *CHEK2*-deficient colonies following 2ME2, ATTM and LCS-1 treatments relative to controls (ns, not significant; ****, *p*-value < 0.0001). Cells were treated for 28-days and the data are presented relative to DMSO treated controls (± SD). **B.** Bar graphs presenting statistically significant decreases in the size of *BLM*- and *CHEK2*-deficient 3D tumor spheres treated with 2ME2, ATTM and LCS-1 relative to controls (ns, not significant; ****, *p*-value < 0.0001). All spheres were treated for 14-days and the data are presented relative to DMSO treated controls (± SD).

To evaluate the long-term (2 and 4 week) effects 2ME2, ATTM and LCS-1 have on cellular growth both colony formation in soft agar and 3D tumor sphere assays were performed. First, standard colony formation assays were conducted 28-days in which DMSO, 2ME2, ATTM or LCS-1 was supplemented into growth media that was replaced every 2-days. In agreement with the above findings, statistically significant decreases were observed for the total number of *BLM*- and *CHEK2*-deficient colonies treated with 2ME2, ATTM and LCS-1 relative to controls (Figure 6A). Next, the efficacy of the compounds was evaluated in 3D tumor sphere models. Tumor spheres were generated (see Materials and Methods) and treated with compounds or vehicle control, with media (with and without compounds) replaced every 2 days. Following a 14-day incubation period, nuclei were counterstained, tumor spheres were imaged and the sizes (diameters) were determined. In general, there was a statistically significant and ~10-fold decrease in the relative size of all *BLM*- and *CHEK2*-deficient tumor spheres treated with 2ME2, ATTM and LCS-1 relative to controls (Figure 6B). Collectively, these data indicate that 2ME2, ATTM, and LCS-1 treatments decrease the total number of *BLM*- and *CHEK2*-deficient colonies, and also the sizes of 3D tumor spheres, suggesting each is a strong lead candidate therapeutic compound.

## DISCUSSION

In this study, we evaluated the ability of SL interactions initially identified in yeast to predict evolutionarily conserved interactions within a human cell context. Specifically, we sought to determine the ability of SOD1 silencing and inhibition to exploit defects within two genes, *BLM* and *CHEK2*, which normally encode functions within the HDR pathway. Using two isogenic cell models we demonstrate that SOD1 silencing induces preferential killing within *BLM*- and *CHEK2*-deficient cells, and show that these SL interactions are conserved in an unrelated and immortalized cell type. We further show that three chemicals predicted to functionally substitute for SOD1 silencing also induce preferential killing within *BLM-* and *CHEK2*-deficient cells in short (5-day), moderate (14-day) and long-term (28-days) assays in both 2D culture and 3D tumor models. Finally, we show that each compound induces ROS, is associated with increases in DNA DSBs, and correlates with increases in apoptosis within *BLM*- and *CHEK2*-deficient cells. Accordingly, this study identifies two evolutionarily conserved SL interactions, namely *BLM SOD1* and *CHEK2 SOD1*, and defines SOD1 as a novel candidate drug target in cancers harboring *BLM* and *CHEK2* defects. Finally, our data identify 2ME2, ATTM and LCS-1 as lead candidate compounds warranting further pre-clinical study. Collectively, this study underscores the utility of SL datasets generated in model systems (e.g. budding yeast) to uncover evolutionarily conserved and cancer-relevant interactions that will assist in cancer drug target discovery.

SOD1 is highly conserved throughout evolution [33] and its central role in the removal of superoxide radicals and the prevention of excessive oxidative DNA damage is well established in model organisms and humans [34, 35]. SOD1 is a non-essential gene in yeast [36] and mice [37], and the transient nature of treatments is predicted to have minimal impact on normal human cells. Moreover, the EC_50_ values employed in this study are specific to the *BLM*- and *CHEK2*-deficient cells, and are significantly lower than those of the controls (54- to 3, 150-fold). With respect to the compounds, ATTM has been employed for unrelated pathologies, while 2ME2 and LCS-1 remain relatively unexplored. For example, ATTM was originally employed to treat copper poisoning in livestock [38], while in humans it is commonly used to treat Wilson's disease, a neuropsychiatric disorder resulting from Cu^2+^ accumulation. Most recently, ATTM is under investigation for its anti-angiogenic potential based on its ability to prevent endothelial cell homing, cell motility and invasiveness [39–43]. In 2011, Somwar *et al* [21] identified LCS-1 as a selective SOD1 inhibitor and showed it induced killing in lung cancer cells. Most recently, several studies have begun to explore the susceptibility of vascular endothelial cells to ROS [44–47], suggesting SOD1 inhibitors and ROS inducers may hold additional potential as anti-angiogenic agents. In the current study, we repurposed these chemicals to induce SL killing within two specific genetic contexts (i.e. somatic *BLM*- or *CHEK2*-deficiencies). Thus, it is possible that 2ME2, ATTM or LCS-1 treatments in *BLM*- or *CHEK2*-deficient/defective tumors may induce anti-angiogenic effects that will synergize with the SL interactions to prevent tumor vascularization while inducing SL killing.

Although the primary goal of this work is to exploit somatic mutations in *BLM* and *CHEK2*, it is possible that 2ME2, ATTM and LCS-1 may also be effective in familial cancers, in much the same manner that PARP1 inhibitors are being evaluated in the context of familial breast and ovarian cancers harboring inherited defects in *BRCA1/2*. In a familial cancer context, all individuals are expected to inherit a single wile-type allele and a mutant allele, and it is the subsequent loss of heterozygosity that contributes to the development of familial tumors. Thus, it may be possible to selectively target familial cancers as the non-cancerous (i.e. normal) cells are expected to harbor a single wild-type copy of *BLM* or *CHEK2*, and produce sufficient protein to render these cells resistant to a SL attack.

Over the five past years, there have been numerous successful approaches designed to identify SL interactors of cancer-associated genes, with the ultimate goal of identifying novel drug targets. In 2013, Vizeacoumar *et al* [48] performed a genome-wide screen to uncover negative genetic interactions (*i.e*. SL interactors) across a set of isogenic cancer cell lines, including the *BLM*-deficient cells employed in the current study. Interestingly, they did not identify *SOD1* as a lead SL interactor, which we attribute to two fundamental differences between our studies. First, Vizeacoumar and colleagues employed a microarray-based approach that is based on the loss of the bar-coded shRNAs within a population of cells, which may not have occurred within the timeframe of their experiment. Second, and perhaps most likely, although SOD1-induced killing is robust within our study, it may not have fallen below the operational threshold required for subsequent validation within their study. Nevertheless, our data complement their findings, and expands the number of validated SL interactors and drug targets of *BLM*-deficient cells.

The current study provides the first evidence that the *dun1 sod1* SL interaction first identified in yeast [27], is evolutionarily conserved in a human cancer context. More specifically, we identify SOD1 as the first SL interactor and drug target capable of exploiting genetic defects in *CHEK2*. CHEK2 is a particularly attractive and unexplored gene to examine in direct SL tests as it has an established role in HDR [49], it is normally required for chromosome stability [50, 51], and mutations are associated with both familial and sporadic cancers [52–55]. However, *CHEK2* is also of interest as a therapeutic target that can potentiate the cytotoxic effects associated with DNA damage, radiation or chemotherapeutic compounds including camptothecin [56], PARP inhibitors or doxorubicin [57]. With the knowledge that CHEK2 regulates TP53-mediated apoptosis [58], another therapeutic strategy has been to target CHEK2 activity to sensitize *TP53*-deficient cells to compounds that induce genotoxic stress [59, 60]. In 2009, Jiang *et al* [60] demonstrated that following CHEK2 depletion, *TP53*-deficient cells were sensitized to doxorubicin, while the *TP53*-proficient cells were resistant. Although speculative, we predict that the simultaneous depletion of CHEK2 in combination with SOD1 silencing or 2ME2, ATTM or LCS-1 treatments may further exacerbate the sensitivity of TP53-deficient cells to doxorubicin. Thus, targeting SOD1 may have additional therapeutic implications beyond the *BLM* and *CHEK2* genetic contexts explored in the current study.

A major goal of the current study was to determine whether SOD1 was a shared SL interactor for genes involved in HDR in humans. Beyond the *BLM* and *CHEK2* contexts examined in the current study, SOD1 may represent a common therapeutic target capable of exploiting many additional genetic defects. Extensive SL data generated in budding yeast [27, 61, 62] have shown that members of a given biological pathway (e.g. HDR) frequently share SL interactors. We previously showed that *RAD54B*, a gene that encodes a helicase functions within the HDR pathway is also SL with *SOD1* [20]. While *RAD54B* is somatically altered in ~3.3% of CRCs [3, 4, 6], *BLM* and *CHEK2* are mutated in up to 4.1% [6] and 6.9% [3, 4, 63], respectively. Thus, mutations in these three genes alone account for ~14.4% of all CRCs, which amounts to ~19,000 Americans annually who may be potentially responsive to a SOD1-directed therapy. Due to the evolutionarily conserved nature of these SL interactions, it appears that SOD1 is a putative therapeutic hub that may harbor addition SL interactions with other HDR genes. Although speculative, this list could include other key HDR genes including *ATM*, *BRCA1/2*, *MRE11*, *RAD50*, *NBS1* or *RAD51*, which are also mutated in CRC and many other tumor types. Furthermore, somatic mutations of *SOD1* are rare in cancer, and those few that do occur are mutually exclusive of those occurring for *RAD54B*, *BLM* and *CHEK2* [3, 4, 6, 64–67]. These observations suggest that mutations leading to a loss of function for SOD1 are not tolerated in cancer cells harboring pre-existing mutations within *RAD54B*, *BLM* or *CHEK2*, and further support SOD1 as a strong candidate therapeutic target. Finally, SOD1 may also represent an attractive therapeutic target beyond CRC. Although *RAD54B*, *BLM* and *CHEK2* genes are mutated in CRC, they are also mutated in numerous additional cancers including pancreatic (~16.5% collectively), prostate (~16.7%), melanoma (~15.1%) and endometrial (~8.8%). Thus, studies aimed at exploring SOD1 as a candidate drug target in other HDR-defective gene contexts, and tumor types are highly warranted.

## SUPPLEMENTARY FIGURES AND TABLES


