# Supplementary material for: Targeting SOD1 induces synthetic lethal killing in BLM- and CHEK2-deficient colorectal cancer cells
Source: Oncotarget. 2015 Jul 31;6(29):27907–22. doi: 10.18632/oncotarget.4875 (PMC4695034; doi:10.18632/oncotarget.4875)
Supplement: Supplementary file 1 [file oncotarget-06-27907-s001.pdf]

SUPPLEMENTARY FIGURES AND TABLES

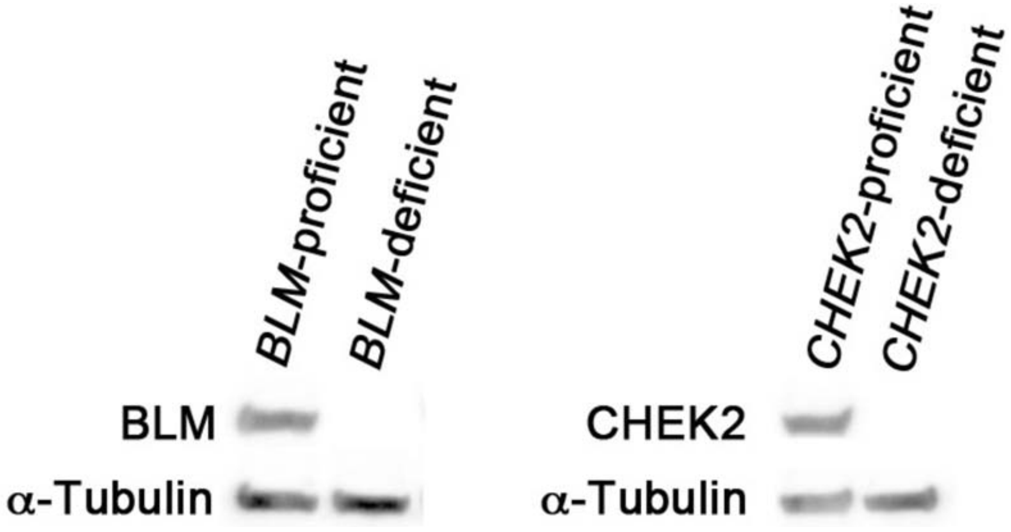

Supplementary Figure S1: Confirming BLM and CHEK2 expression levels. Western blots depicting the expression levels of BLM (left) and CHEK2 (right) within the *BLM*-deficient and *CHEK2*-deficient cell lines, respectively, relative to control HCT116 cells; α-Tubulin serves as the loading control.

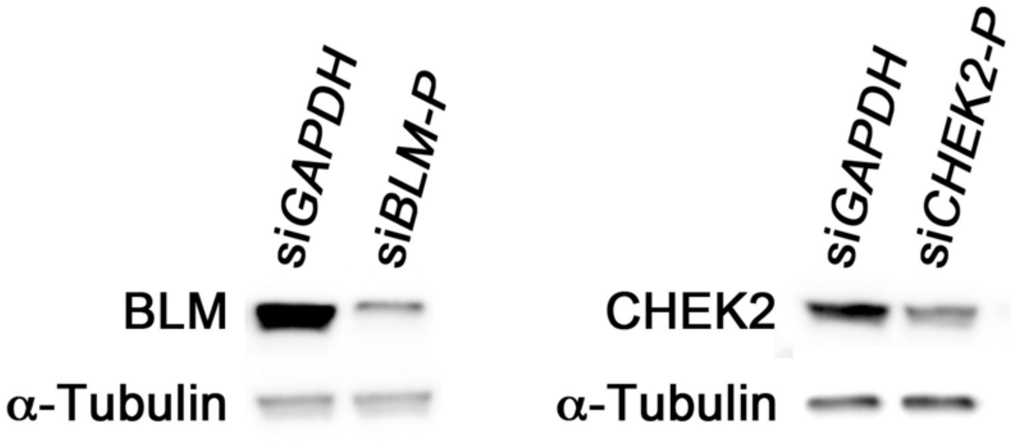

Supplementary Figure S2: Silencing of BLM and CHEK2 in HCT116 cells. Western blots presenting BLM (left) and CHEK2 (right) expression levels following siRNA-based silencing (siBLM-P, siCHEK2-P, and siGAPDH [negative control]) in HCT116 cells; α-Tubulin serves as a loading control.

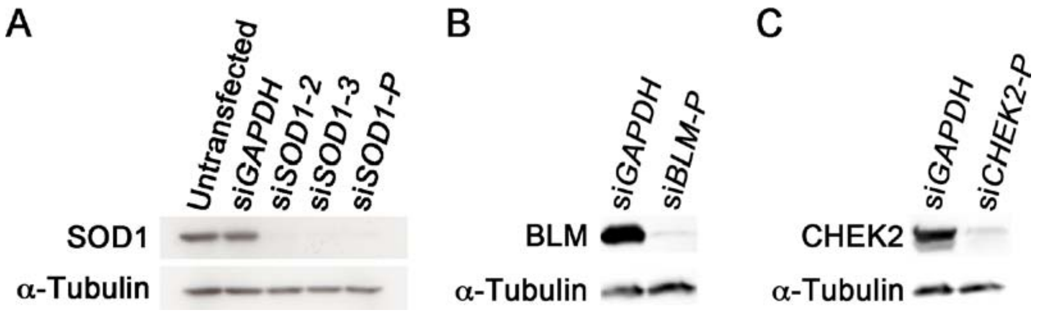

Supplementary Figure S3: Evaluating protein expression levels in hTERT cells. A. Western blot demonstrating SOD1 silencing with either individual (siSOD1–2 and siSOD1–3) or pooled (siSOD1-P) siRNA duplexes in hTERT cells relative to controls (untransfected and siGAPDH); α-Tubulin serves as a loading control. **B.** Western blot depicting BLM expression levels following siRNA-based silencing in hTERT cells. **C.** Western blot presenting CHEK2 expression levels following silencing in hTERT cells.

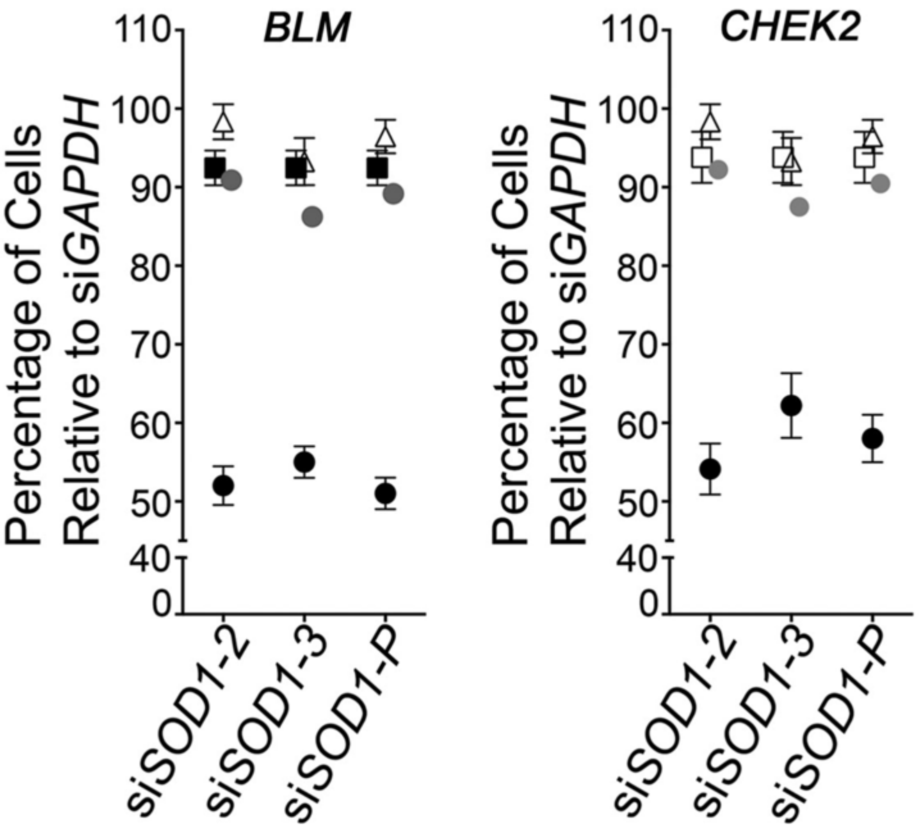

Supplementary Figure S4: BLM and CHEK2 are SL with SOD1 in hTERT cells. Graphs depicting the SL interaction observed following simultaneous silencing of BLM (left) or CHEK2 (right) with SOD1 in hTERT cells. Presented are the mean normalized percentages (± SD) for the individual silencing of either BLM (solid squares) or CHEK2 (open squares) and SOD1 (open triangles), and the expected value (grey circles) determined for the dual combined siRNAs as calculated using a multiplicative model. Solid circles identify the actual observed values for the simultaneous dual silencing (i.e. BLM and SOD1, or CHEK2 and SOD1) and are consistently lower than the corresponding expected values.

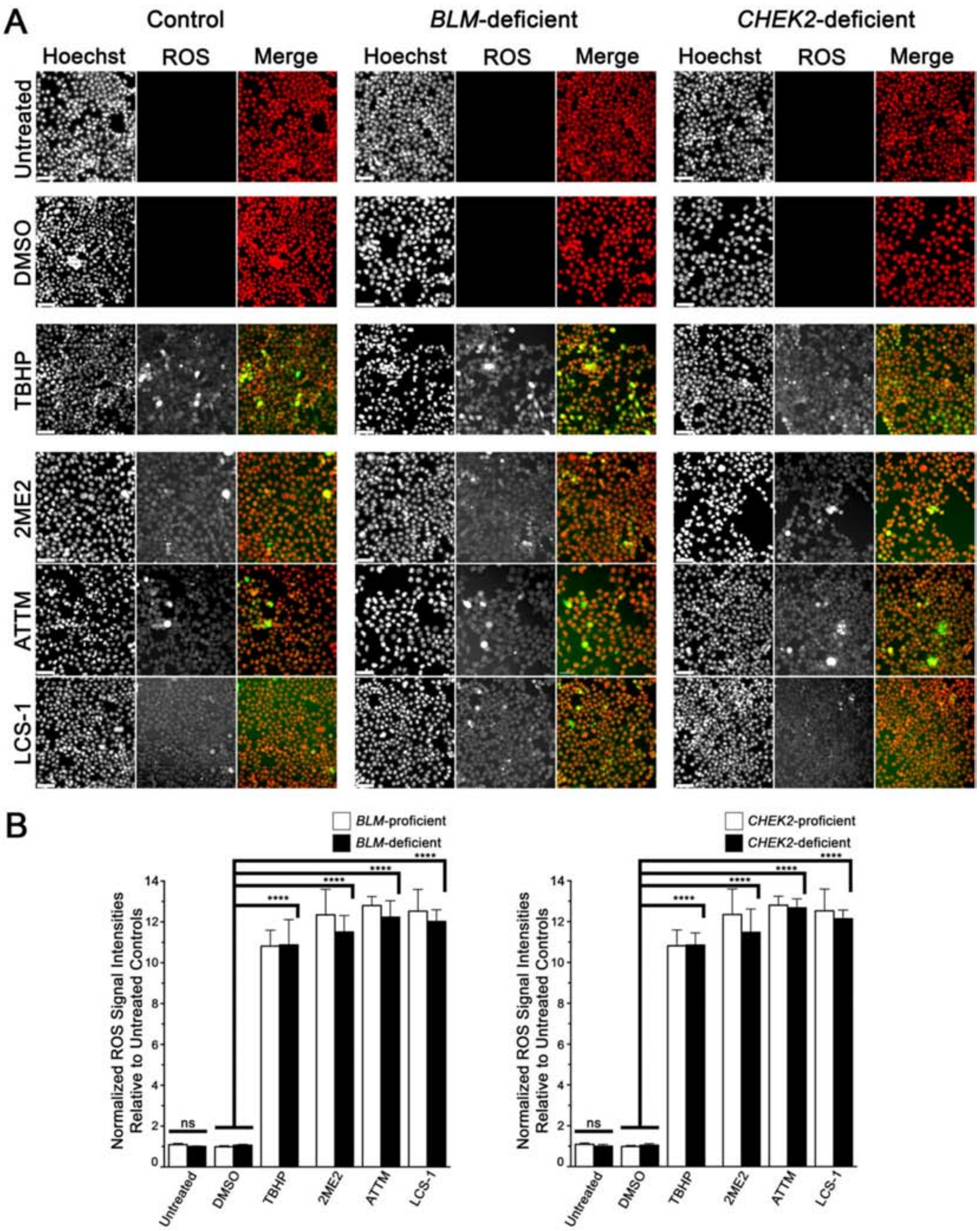

Supplementary Figure S5: 2ME2, ATTM and LCS-1 induce increases in ROS. A. Representative low-resolution images (10×) presenting the qualitative differences in ROS signal intensities observed within control, *BLM*- and *CHEK*-2-deficient cells treated with 2ME2, ATTM and LCS-1 for 6 h. Tert-butyl hydroperoxide (TBHP) is an inducer of ROS and a positive control, while untreated and DMSO treated cells serve as negative controls. ROS was detected using the Image-IT Live Green ROS detection kit and nuclei were counterstained with Hoechst. All images were acquired using identical exposure times at each wavelength. Hoechst and ROS are pseudo-colored red and green, respectively, within the merged images. Scale bars represent 100 µm. Note the visually striking increases in ROS signal intensities within all cells treated with 2ME2, ATTM and LCS-1 relative to the respective controls. **B.** Bar graphs depicting the mean normalized ROS signal intensities (± SD) within control, *BLM*- (left) and *CHEK2*-deficient (right) cells treated with DMSO, TBHP, 2ME2, ATTM or LCS-1. All data are presented relative to the DMSO treated controls as determined by semi-quantitative analysis performed on raw, unprocessed images. Note the statistically significant increases in ROS signal intensities within the *BLM*- and *CHEK2*-deficient cells relative to controls (****, *p*-value < 0.0001; ns = not significant).

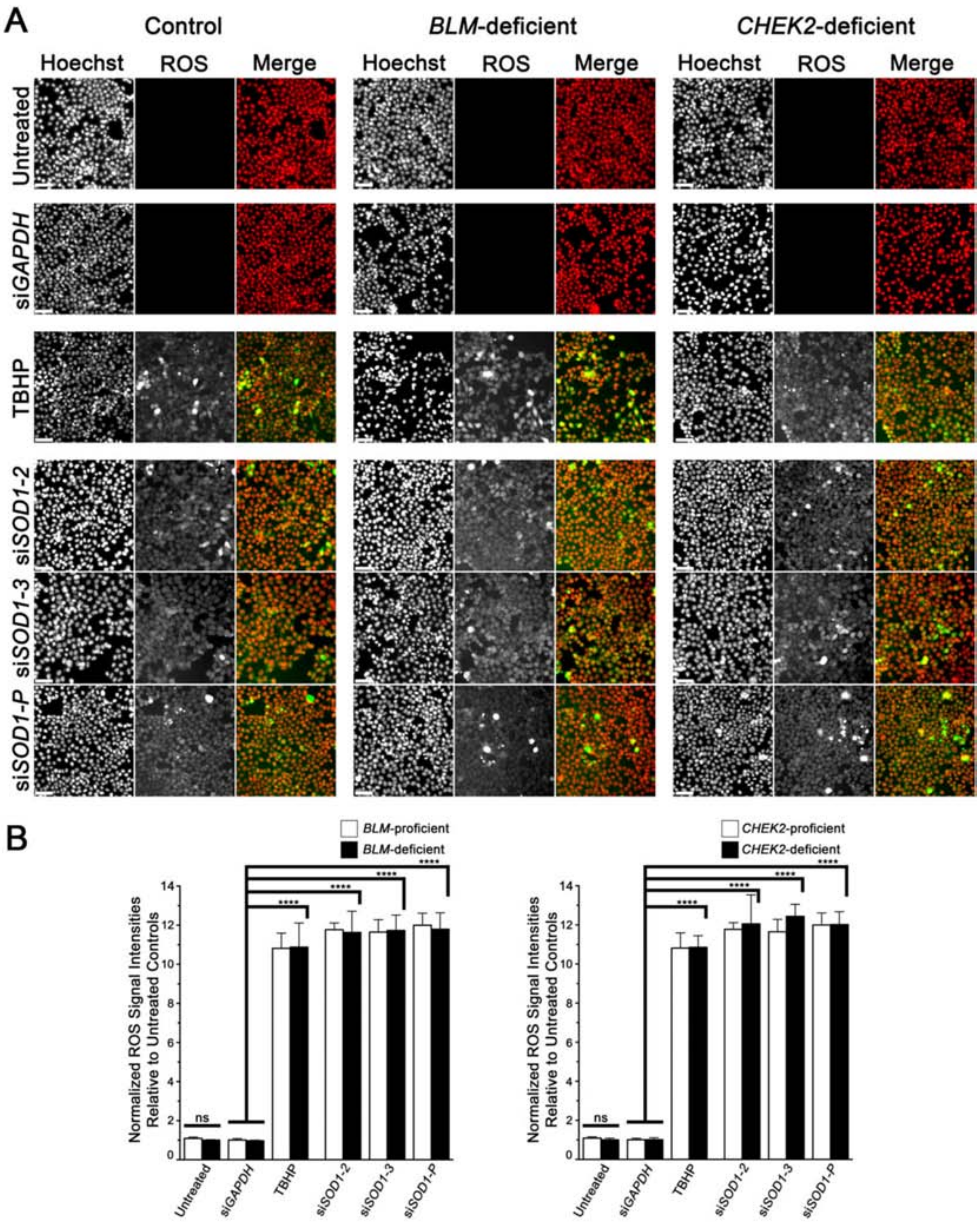

Supplementary Figure S6: Diminished expression of SOD1 induces ROS production. A. Representative low-resolution images (10×) presenting the qualitative differences in ROS signal intensities observed within control, *BLM*- and *CHEK*-2-deficient cells treated with either siRNAs targeting GAPDH (siGAPDH) or SOD1 (siSOD1–2, siSOD1–3 and siSOD1-P). TBHP is an inducer of ROS and a positive control, while untreated and GAPDH silenced cells serve as negative controls. ROS was detected using the Image-IT Live Green ROS detection kit and nuclei were counterstained with Hoechst. All images were acquired using identical exposure times at each wavelength. Hoechst and ROS are pseudo-colored red and green, respectively, within the merged images. Scale bars represent 100 µm. Note the visually striking increases in ROS signal intensities within all cells treated with siRNAs targeting SOD1 relative to the respective controls. **B.** Bar graphs depicting the mean normalized ROS signal intensities (± SD) within control, *BLM*- (left) and *CHEK2*-deficient (right) cells silenced for SOD1 or GAPDH or relevant controls. All data are presented relative to the GAPDH silenced controls. As above, raw, unprocessed images were used to determine and quantify ROS signal intensities. Note the statistically significant increases in ROS signal intensities within the *BLM*- and *CHEK2*-deficient cells relative to controls (****, p-value < 0.0001; ns = not significant).

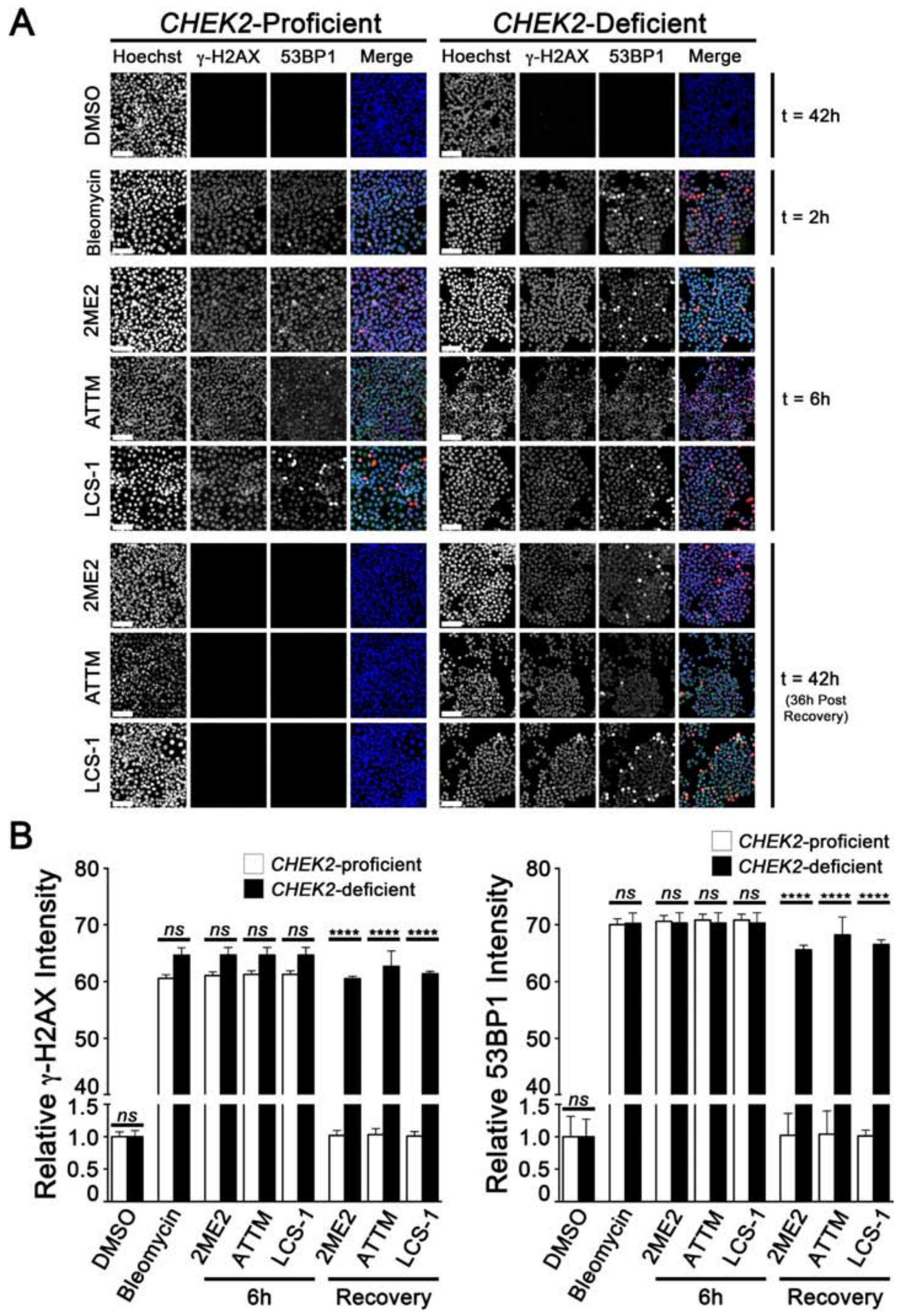

Supplementary Figure S7: DNA DSBs persist in CHEK2-deficient cells treated with 2ME2, ATTM and LCS-1. **A.** Representative low-resolution (10×) images presenting the qualitative changes in γ-H2AX and 53BP1 signal intensities within control (left) and *CHEK2*-deficient cells (right) treated with DMSO, bleomycin (positive control), 2ME2, ATTM, or LCS-1. Cells were imaged after 2 h (t = 2 h; bleomycin) or 6 h (t = 6 h; DMSO, 2ME2, ATTM and LCS-1) treatments, or following treatment, washout and a 36 h recovery phase (t = 42 h). Nuclei were counterstained with Hoechst, and images were acquired using identical exposure times at each wavelength so that qualitative and quantitative analyses could be performed. Hoechst, γ-H2AX and 53BP1 are pseudo-colored blue, green, and red, respectively, within the merged images. Scale bars represent 100 µm. Note the persistence of γ-H2AX and 53BP1 signal intensities within the *CHEK2*-deficient cells following washout and recovery relative to controls. **B.** Graphs presenting the mean normalized γ-H2AX (left) and 53BP1 (right) signal intensities (± SD) within control and *BLM*-deficient cells treated with DMSO, bleomycin, 2ME2, ATTM, or LCS-1 or following washout and a 36 h recovery phase (t = 42 h). All data are presented relative to the DMSO-treated controls. Raw, unprocessed images were used to determine and quantify γ-H2AX and 53BP1 signal intensities. Note the persistence and statistically significant differences observed for γ-H2AX and 53BP1 following washout and recovery within the *CHEK2*-deficient cells relative to controls (ns, not significant; ****, p-value < 0.0001).

Supplementary Table S1: Antibodies employed and their working concentrations.

			Working Dilution	
**Epitope**	**Vendor**	**Catalog No.**	**WB[A]**	**IIF[B]**
BLM	Abcam	ab476	1:1, 000	N/A[C]
CHEK2	Abcam	ab109413	1:5, 000	N/A
SOD1	Abcam	ab13498	1:2, 500	N/A
α-Tubulin	Abcam	ab7291	1:10, 000	N/A
γ-H2AX	Abcam	ab18311	N/A	1:1, 000
53BP1	Abcam	ab70323	N/A	1:500
Cleaved Caspase 3	Abcam	ab13847	N/A	1:1, 500
Rabbit IgG-HRP	Jackson ImmunoResearch	111-035-006	1:15, 000	N/A
Mouse IgG-HRP	Jackson ImmunoResearch	115-035-146	1:10, 000	N/A
Goat anti-Rabbit Alexa488	Molecular Probes	A-11034	N/A	1:200
Goat anti-Mouse Cy3	Molecular Probes	A-10521	N/A	1:200

[A]WB; Western blot
[B]IIF; Indirect immunofluorescence
[C]N/A; not applicable

Supplementary Table S2: Student's *t*-tests reveal statistically significant decreases in *BLM*- and *CHEK2*-deficient cell numbers following SOD1 silencing[A].

		Mean Cell Number ± SD[C]			*p*-value[D]	
**siRNA Treatment**	**N[B]**	**HCT116 (Control)**	***BLM*-deficient**	***CHEK2*-deficient**	***BLM***	***CHEK2***
siGAPDH	6	4390 ± 471.1	4290 ± 125.8	4391 ± 253.3	ns	ns
siSOD1-P	6	4246 ± 74.4	1217 ± 335.5	1075 ± 214.3	<0.0001	<0.0001
siSOD1-2	6	4353 ± 157.1	1306 ± 399.2	1054 ± 160.5	<0.0001	<0.0001
siSOD1-3	6	4244 ± 108.2	1299 ± 307.7	1706 ± 205.6	<0.0001	<0.0001

[A]Only a single representative example from an experiment conducted in sextuplet is shown. Each experiment was conducted two additional times with similar results.
[B]N; number of assay wells analyzed
[C]SD; standard deviation
[D]*p*-value calculated for either the *BLM*- or *CHEK2*-deficient condition relative to the corresponding control cells (HCT116 [*BLM*- and *CHEK2*-proficient]), ns; not significant

Supplementary Table S3: Dual silencing experiments validate both *BLM* and *CHEK2* are SL with *SOD1* in HCT116 cells.

siRNA Treatment	N[A]	Mean ± SD[B]	Relative Percentage of Cells (%)[C]		Relative Percent Change (%)[E]
			Observed	Expected[D]	
siGAPDH	6	4697 ± 98.9	100.0	N/A	N/A
siBLM	6	4442 ± 89.7	94.6	N/A	N/A
siCHEK2	6	4243 ± 132.6	90.3	N/A	N/A
siSOD1-P	6	4376 ± 103.8	93.2	N/A	N/A
siSOD1-2	6	4352 ± 63.2	92.7	N/A	N/A
siSOD1-3	6	4449 ± 168.1	94.7	N/A	N/A
siBLM + siSOD1-P	6	2981 ± 49.3	60.1	88.1	31.8
siBLM + siSOD1-2	6	2877 ± 134.6	59.6	87.6	32.0
siBLM + siSOD1-3	6	2966 ± 98.7	58.2	89.6	35.0
siCHEK2 + siSOD1-P	6	2821 ± 119.3	63.5	84.1	24.5
siCHEK2 + siSOD1-2	6	2799 ± 84.6	61.2	83.7	26.9
siCHEK2 + siSOD1-3	6	2733 ± 78.1	63.2	85.6	26.2
siPLK1	6	222 ± 196.3	4.7	N/A	N/A

[A]N; number of wells analyzed per condition
[B]Mean number of cells imaged/well ± standard deviation (SD)
[C]Expressed relative to the negative control (siGAPDH)
[D]Calculated by multiplying the Observed percentages for the two corresponding individual siRNA treatments. N/A; not applicable
[E]Calculated as 100 × (1 – [Observed/Expected])

Supplementary Table S4: Dual silencing experiments demonstrating *BLM* and *CHEK2* are SL with *SOD1* in hTERT cells.

siRNA Treatment	N[A]	Mean ± SD[B]	Relative Percentage of Cells (%)[C]		Relative Percent Change (%)[E]
			Observed	Expected[D]	
siGAPDH	6	2356 ± 114.6	100.0	N/A	N/A
siBLM	6	2178 ± 163.4	92.5	N/A	N/A
siCHEK2	6	2210 ± 74.8	93.8	N/A	N/A
siSOD1-P	6	2272 ± 68.1	96.5	N/A	N/A
siSOD1-2	6	2316 ± 118.3	98.3	N/A	N/A
siSOD1-3	6	2197 ± 141.2	93.3	N/A	N/A
siBLM + siSOD1-P	6	1202 ± 131.9	51.0	89.2	42.8
siBLM + siSOD1-2	6	1225 ± 144.8	52.0	90.9	42.8
siBLM + siSOD1-3	6	1296 ± 129.8	55.0	86.2	36.2
siCHEK2 + siSOD1-P	6	1367 ± 116.7	58.0	90.5	35.9
siCHEK2 + siSOD1-2	6	1271 ± 177.9	54.1	92.2	41.3
siCHEK2 + siSOD1-3	6	1465 ± 77.6	62.2	87.5	28.9
siPLK1	6	169 ± 108.7	7.3	N/A	N/A

[A]N; number of wells analyzed per condition
[B]Mean number of cells imaged/well ± standard deviation (SD)
[C]Expressed relative to the negative control (siGAPDH)
[D]Calculated by multiplying the Observed percentages for the two corresponding individual siRNA treatments. N/A; not applicable
[E]Calculated as 100 × (1 – [Observed/Expected])
